# Colchicine: Isolation, LC–MS QTof Screening, and Anticancer Activity Study of *Gloriosa superba* Seeds

**DOI:** 10.3390/molecules24152772

**Published:** 2019-07-30

**Authors:** Acharya Balkrishna, Subrata K. Das, Subarna Pokhrel, Alpana Joshi, Laxmi, Sudeep Verma, Vinai K. Sharma, Vinamra Sharma, Niti Sharma, C. S. Joshi

**Affiliations:** Drug Discovery & Development Division, Patanjali Research Foundation (Trust), Near Patanjali Yogapeeth-I, Haridwar, Pin- 249405 Uttarakhand, India

**Keywords:** *Gloriosa superba*, super critical fluid (CO_2_) extraction (SCFE), colchicine, cytotoxicity, cell cycle

## Abstract

Colchicine was extracted from *Gloriosa superba* seeds using the Super Critical Fluid (CO_2_) Extraction (SCFE) technology. The seeds were purified upto 99.82% using column chromatography. Colchicine affinity was further investigated for anticancer activity in six human cancer cell lines, i.e., A549, MCF-7, MDA-MB231, PANC-1, HCT116, and SiHa. Purified colchicine showed the least cell cytotoxicity and antiproliferation and caused no G2/M arrest at clinically acceptable concentrations. Mitotic arrest was observed in only A549 and MDA-MB231 cell lines at 60 nM concentration. Our finding indicated the possible use of colchicine at a clinically acceptable dose and provided insight into the science behind microtubule destabilization. However, more studies need to be conducted beforethese findings could be established.

## 1. Introduction

Traditionally, *Gloriosa* is used in the treatment of various clinical conditions like inflammation, edema, rheumatism, skin infections, arthritis, alopecia, and many others [1,2,3].Colchicine is an alkaloid present in the seeds of *Colchicum autumnale* L. and *Colchicum luteum L.*, as well as in tubers of *Gloriosa superba* L. (family Liliaceae) [4]. *Gloriosa* grows throughout the tropical region in India and is a well-known source of colchicine. Its use is associated with a decreased risk of incidence in all types of cancers [5,6]. On the basis of dry mass, *Colchicum* and *Gloriosa* contains 0.62% to 0.9% of colchicine [7,8]. The higher content of colchicine in *Gloriosa* than in *Colchicum* makes it a commercially viable source of this medicine [9,10]. Several techniques have been developed to extract colchicine, and among these ‘soxhlet and solid–liquid extraction’ are the most commonly used [11,12]. Extraction of colchicine from *C. autumnale* using methanol [6], and from *Gloriosa* seeds by methanol [13] and ethanol [14] has also been reported.

In recent years, the use of Super Critical Fluid (CO_2_) Extraction (SCFE) for extraction of phytochemicals has been fast increasing. SCFE is superior when compared to the traditional solvent extraction because the final extract is inexpensive and free from residual solvents. Additionally, SCFE extraction with CO_2_ is considered to be an environment-friendly technology [14,15,16,17,18]. The SCFE is also used along with a co-solvent, which enhances the extraction efficiency [15,19,20,21,22,23,24,25]. Colchicine was studied for its tubulin inhibitory activity and was found to bind at a location where it prevents curved tubulin [26]. Colchicine is used as a remedy for treating gout [27], and as an antidote for snake bite [28] and Familial Mediterranean Fever [29,30]. Due to its significant role in pharmaceuticals, its extraction and production with the lowest impurity content is immensely important.

Colchicine is a well-known potent microtubule targeting agent. It disrupts microtubule functions by binding to various sites on β-tubulin causing mitotic arrest (hyperploidy) [31,32,33,34]. The binding of colchicine to microtubules dissociates them in to tubulin dimers, thereby interfering with the polymerization of tubulin and interrupting microtubule dynamics [35,36]. Colchicine possesses anti-cancerous activity and the mechanism involves inhibition of G1 to M phase progression (G2/M phase arrest) of cell cycle, by disrupting the formation of the mitotic spindle required for mitosis [32,37,38]. Cell cycle arrest regulates cell growth by arresting cell proliferation and induces cell death [39,40]. The rate of mitosis is increased in cancer cells and microtubules formed during mitosis are considered as an ideal target for anticancer drugs [41]. The therapeutic use of colchicine as an anticancer drug is limited due to its toxicity to non-cancerous cell, but oral colchicine is a safe medicine when the dose is used in a controlled way and contraindications are limited [30,41,42].

Colchicine was approved by the Food and Drug Administration (FDA, USA) as a drug for gout, in 2009, and as a single ingredient in the liquid form, in 2019.This has encouraged profound interests in colchicine research and applications. This was the first time colchicine was extracted from Gloriosa seeds using SCFE technology and was standardized for purity, with respect to the reference standards, using High Performance Liquid Chromatography (HPLC). LC–MS quadrupole time-of-flight (QTof) was used for the determination of the seed’s chemical composition [43,44]. Our present study indicated that purified colchicinein low doses has the least cytotoxic effect on human cell lines. Anticancer effect of colchicine was further investigated in six human cancer cell lines.

## 2. Materials and Methods

### 2.1. Plant Material and Chemicals

The *Gloriosa superba* seeds were procured from Salem, Tamil Nadu (India) in February 2016. Liquid CO_2_ of high purity (CAS No. 124-38-9) was procured from the local industrial gas supplier. The solvents used were of LR grade (Qualigens Fine Chemicals, Mumbai, India). Untreated activated charcoal (CAS No. 7440-44-0) (particle size <75 microns) was obtained from Sigma (St. Louis, MO, USA). Alumina neutral activity I-II (Neutral Aluminum oxide active) (CAS No. 1344-28-1) was obtained from Merck India, Mumbai, India. All analytical solvents used were of HPLC grade and were procured from Merck India, Mumbai, India. LC–MS grade solvents were procured from Honeywell, Germany. Colchicine reference standard was procured from Natural Remedies, Bangalore, India.

### 2.2. Super Critical Fluid Extractor

**Figure 1:**
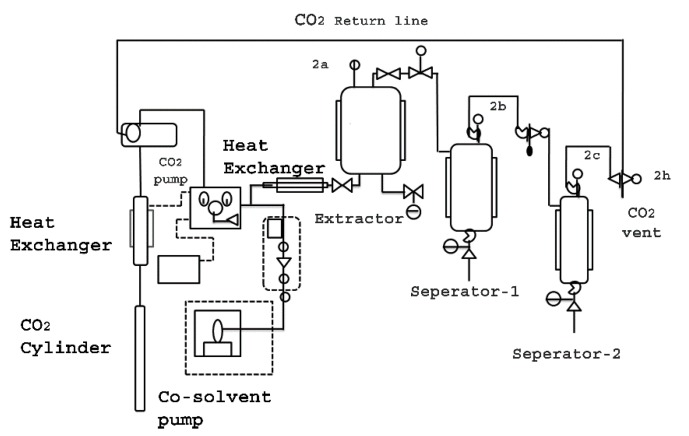
Schematic diagram showing Supercritical Fluid Extractor (2a, 2b, 2c, and 2h are the pressure valves).

Super Critical Fluid (CO_2_) Extractor (Waters Co-Botanical Extraction System 5000) was designed with a 3 Kg loading capacity of the raw material. SCF (CO_2_) extractor was equipped with two separators (collection vessels), using the phenomena of high and low-pressure zones, in the different separators (Figure 1).

### 2.3. Super Critical Fluid (CO_2_) Extraction

*Gloriosa superba* seeds (3 Kg) were extracted using Super Critical CO_2_ extractor, at a temperature of 60 °C and varying pressure of 200–450 bars. The separators were kept at different pressures and temperatures, i.e., 350 bar at 50 °C and 100 bar at 15 °C, respectively. Therefore, the extract was collected in separator1, while wax material, fatty acids and other volatile materials were collected in separator2. The extraction was carried out with liquid CO_2_ with 3% water as co-solvent. Water was used as a co-solvent for extraction to enhance the extraction efficiency and water was removed from the extract by applying low pressure, while it was moved to the separator 2. After placing the basket containing seeds powder inside the extractor, heating/cooling systems were turned ON. The liquid CO_2_ flow rate was kept at a constant 100 g per minute while 3 g of co-solvent water (3% of liquid CO_2_) was used as the extraction media. Once the extraction cylinder temperature reached 60 °C, the pressure valves 2a, 2b, 2c, and 2h were opened. Each extraction was carried out for 3 h. The yield at different extraction pressure conditions was plotted and analyzed (Table 1).

### 2.4. Isolation of Colchicine

The SCF extract from separator 1 was collected and cooled to room temperature. Concentrated extract was dissolved in de-mineralized water (5 L) and filtered using a hiflo-supercell bed. The aqueous layer was partitioned using ethyl acetate (10 L × 2 times). The combined ethyl acetate layer was washed using de-mineralized water (1 L), to remove the phenolic impurities. The ethyl acetate layer was then concentrated and dried at 50 °C, under vacuum. The dried mass (72.8 g) was dissolved in ethanol (200 mL), passed through an activated charcoal (50 g) columnand was eluted using ethanol (2 L). The ethanol elute was concentrated to dryness under vacuum, at 50 °C. Finally, the dried mass (19.1 g), which was found to be 96.7% pure, was dissolved in 200 mL ethyl acetate and was passed through a neutral alumina column (200 g, activity I-II). The column was eluted using ethyl acetate; the combined elute was concentrated to 100 mL, under vacuum, at 40 °C, and was kept for crystallization for 1 h, at room temperature. The crystalline mass was filtered using a Buchner funnel and was dried at 30 °C, under vacuum (1 torr), for 24 h. This yielded 17.83 g (85.2% *w*/*w*) of light yellow crystalline mass containing colchicine 99.82% (*w*/*w*) assay. The analysis was performed using high performance liquid chromatography. 

### 2.5. Preparation of Solutions for HPLC Analysis

A standard stock solution of 473.76 µg/mL was prepared by dissolving 4.8 mg of Colchicine (purity 98.7%) in HPLC grade methanol, in a 10 mL volumetric flask and completely filled.

The sample solution was prepared using 2 g of homogenized Gloriosa seed powder in a 250 mL round bottom flask—50 mL of HPLC grade methanol was added to it and refluxed for 1 h in a water bath and cooled; the solution was then transferred into a 100 mL volumetric flask and HPLC grade methanol was added to it until the volume increased to 100 mL. For the samples of the SCFE extracts, 0.5 g of the extract was dissolved in 50 mL of HPLC grade methanol, while 5.01 mg of purified colchicine was dissolved in 10 mL of HPLC grade methanol.

### 2.6. HPLC Chromatographic Conditions

**Figure 2:**
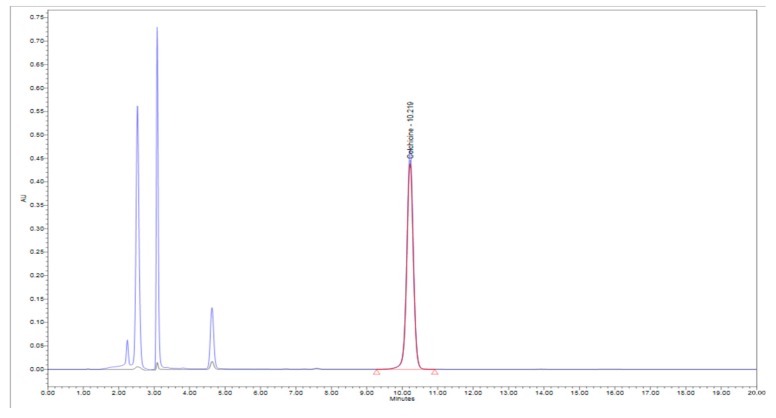
HPLC Chromatograph of the Colchicine standard and the Gloriosa seed extract.

Analysis was performed on Waters HPLC equipped with an on-line degasser, AP 1525 Binary HPLC pump, 2707 Auto Sampler, 2998 Photodiode Array Detector, and Empower-3 software. The XBridge C18 column (250 × 4.6 mm, 5 µm) was maintained at 40 °C throughout the analysis and detection was carried out at 245 nm. The flow rate was maintained at 1 mL/min using isocratic elution of mobile phase Water:Acetonitrile (75:25). A total of 10 µL of the sample solutions was injected for the analysis and the chromatograph was recorded for 20 min (Figure 2).

### 2.7. Preparation of Sample Solution for LC–MS QTof Screening

Liquid chromatography–mass spectrophotometry/quadrupole time-of-flight screening was done for studying the chemical composition of the Gloriosa seeds. A total of 10 mL of methanol:water mixture (80:20) was added to 201.0 mg of powdered *Gloriosa superba* seeds and sonicated for 15 min. This solution was centrifuged for 5 min at 5000 rpm and filtered through a 0.22 µm nylon filter.

### 2.8. LC–MS QTof Conditions

**Figure 3:**
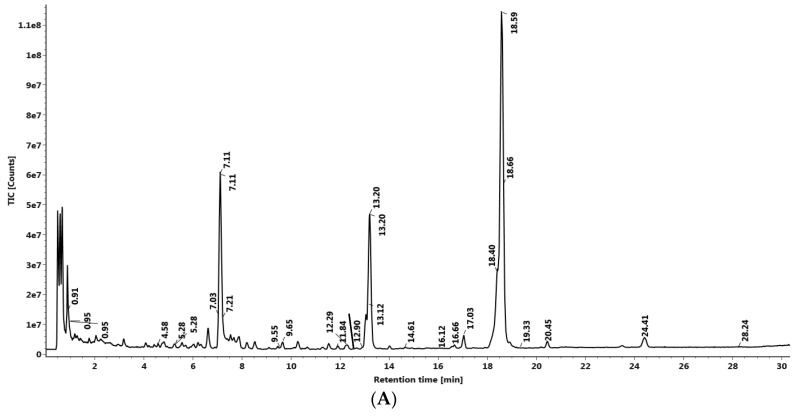

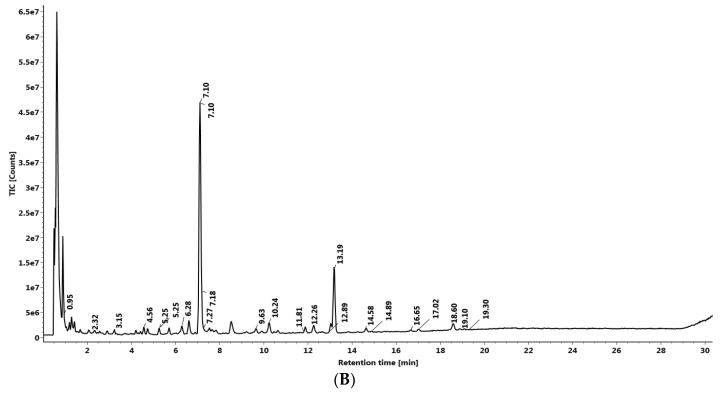
(**A**) UPLC–MS QTof Chromatograph of Gloriosa seed extract in the positive mode. (**B**)UPLC–MS QTof Chromatograph of Gloriosa seed extract in the negative mode.

The LC–MS QTof instrument was equipped with an ESI ion source operating in a positive and negative ion mode. A mass range of 50–1000 Da was set with a 0.2 s scan time. The main working parameters for mass spectrometry were set as follows, ionization type—ESI, mode—MS^E^, acquisition time—30 min, mass range (*m*/*z*)—50–1000 *m*/*z*, low collision energy—6 eV, high collision energy—20–40 eV (ramp), cone voltage—40 V, capillary voltage—1 kV (for positive mode) (Figure 3A), capillary voltage—2 kV (for negative mode) (Figure 3B), source temperature—120 °C, desolvation temperature—500 °C, cone gas flow—50 L/h, desolvation gas flow—800 L/h. Mass was corrected during acquisition, using an external reference (Lock–Spray) consisting of 0.2 ng/mL solution of leucine enkephalin infused at a flow rate of 10 μL/min via a lock–spray interface, generating a reference ion for the positive ion mode [(M + H) + *m*/*z* 556.2766] and for the negative ion mode [(M + H) + *m*/*z* 554.2620] to ensure mass accuracy during the MS analysis. The Lock–Spray scan time was set at 0.25 s with aninterval of 30 s.Analysis was performed on a Waters Xevo G2-XS QTof equipped with Acquity UPLC-I Class and Unifi software. Separation was carried out using Acquity UPLC HSS-T3 column (100 × 2.1 mm, 1.7 µm). The column was maintained at 50 °C throughout the analysis, and sample temperature was kept at 10°C. The elution was carried out at a flow rate of 0.4 mL/min using gradient elution 0.1% formic acid in water (mobile phase A) and 0.1% formic acid in acetonitrile (mobile phase B). Solvent gradient program was 95%–85% of the mobile phase A during 0–4 min, 85%–80% A during 4–15 min, 80%–75% A during 15–20 min, 75% A during 20–28 min, 75%–60% A during 28–31 min, 60%–10% A during 31–32 min, 10% A during 32–34 min, 10%–95% A during 34–35 min, followed by 95% A during 35–37 min. A total of 1µl of the test solution was injected for the screening and the chromatograph was recorded for 30 min.

### 2.9. Cell Lines and Culture Condition

The six human cancer cell lines, lung cancer (A549), breast cancer (MCF-7 and MDA-MB231), pancreatic carcinoma (PANC-1), colon carcinoma (HCT116), and cervical cancer (SiHa) were obtained from National Centre for Cell Science (NCCS) Pune, India. Cell lines were cultured in a DMEM medium supplemented with 10%FBS and 1X antibiotic. The cultured cells were seeded in 24-well plate at a density of 0.05 × 10^6^ and grown overnight at 37 °C in a humidified atmosphere of 5% CO_2_. 

### 2.10. Cell Cytotoxicity Assay

Cell lines were seeded in 96-well plates (1 × 10^4^ cells/mL). After 24 h incubation, the cells were treated with different concentrations of colchicine and Gloriosa extract (concentration was calculated on the colchicine content basis) 0–2560 nM for 48 h. Cells were washed with PBS (twice), fixed with 10% formalin for 30 min and washed with H_2_O. The cells were then stained with 0.5% (*w*/*v*) crystal violet (25% (*v*/*v*) methanol) for 25 min. After washing with water until no color was eluted, the cells were dried overnight. Crystal violet [45] was then eluted with 10% acetic acid and the absorbance of the solution was measured using a multi-mode plate reader (Perkin Elmer, EnVision) at a wavelength of 600 nm.

### 2.11. Cell Migration Assay

Cells were seeded at a density of 0.05 × 10^6^ cells/mL on a 24-well plate. The confluent cell monolayer was scratched with a pipette tip, the floating cells were removed and treated with a varying dose of colchicine (0–60 nM) and then imaged at 0, 24, 48, and 72 h post-scratch. Three independent areas per treatment group were recorded, averaged for each time point, and plotted against time to calculate the rate of migration. The cell-free areas were measured using Carl Zeiss AxioVision SE64 Rel. 4.9.1 software (Germany). 

### 2.12. Cell Cycle Analysis by Flow Cytometry 

The cells were grown in 24-well plate for 24 h to study the effect of colchicine on cell cycle and were then treated with colchicine of different concentrations (0, 2.5, 5, 10, 20, 40, and 60 nM). The cells were then incubated overnight at 37 °C in a humidified atmosphere of 5% CO_2_. The cells treated with colchicine were harvested and fixed in ice cold 70% ethanolin ice, for 1 h. Then, the cells were incubated for 30 min at 37 °C in a PBS solution containing 1 mg/mL RNaseA and 1 µg/mL propidium iodide (PI) solution. The cell cycle analysis was performed using Amnis Image Stream flow cytometer (Millipore, United States) and the data were analyzed based on 8000 events recorded using IDEAS image Stream Analysis Software.

### 2.13. Statistical Analysis 

Experimental values are expressed as mean ± standard deviation of at least two experiments, in triplicates. The level of *p* ≤ 0.05 was used as the criterion for statistical significance.

## 3. Results 

### 3.1. Super Critical Fluid Extraction

Colchicine (a pharmacologically active constituent that has for centuries been used in acute gout arthritis [27]) was isolated on acommercial scale with high purity99.82%, (*w*/*w*), quantified by HPLC.

This was the first time colchicine was extracted from *Gloriosa* seeds using Super Critical Fluid Extractor (SCFE) (Figure 1). The extractor was loaded with 3 Kg of *Gloriosa* seeds powder containing 0.70% colchicine content for every batch. Liquid CO_2_ with 3% water was used as the co-solvent in the extraction medium, at varying pressure, i.e., 200, 250, 300, 350, 400, and 450 bar, at a temperature of 60 °C, which was kept constant for two hours. The study showed that the extraction at a pressure of 400 bar gave an optimum yield of 93.6% (*w*/*w*), on the basis of the colchicine content, i.e., 27% colchicine content (*w*/*w*) (Table 1). 

### 3.2. Isolation and Quantification of Colchicine

The isolation methodology involved column chromatographic separation in which the charcoal column separated most of the impurities, whereas the assay of 99.82%with a final yield of 85.2% was achieved when passed through the alumina columnand the final product was analyzed by HPLC (Figure 2). Structural elucidation was carried out by ^1^H NMR and ^13^C NMR spectroscopy, using JEOL 400 MHz instrument (USA).

### 3.3. LC–MS QTof Screening of Gloriosa Seeds 

Screening of the phytochemical composition of the Gloriosa seeds was analyzed using UPLC–MS QTof (Xevo G2-XS QTof, Waters with Acquity UPLC-I Class), where 32 compounds were identified in the positive mode of ionization (Figure 3A, Table 2) and 10 other constituents were identified in the negative mode of ionization (Figure 3B, Table 2), among the total 42 identified compounds.

### 3.4. Effect of Colchicine on Cell Viability

**Figure 4:**
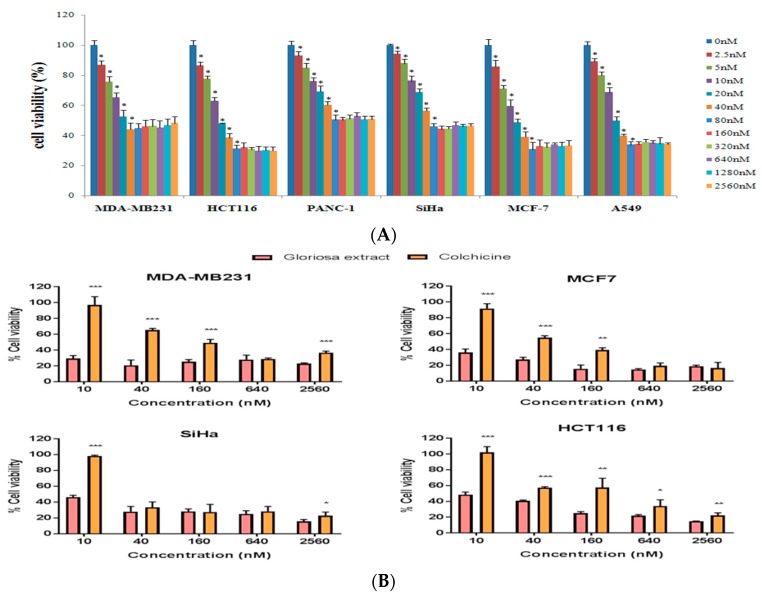
(**A**)Effect of colchicine on cell viability in six human cancer cell lines (MDA-MB231, HCT116, PANC-1, SiHa, MCF-7, and A549) based on Crystal Violet Assay. (**B**)The comparative effect of colchicine and Gloriosa extract (Colchicine content in Gloriosa extract) on cell viability against four human cancer cell lines (MDA-MB231, HCT116, SiHa, and MCF-7).

Effect on cell viability was evaluated using crystal violet staining assay [45] against six human cancer cell lines (A549, HCT116, MCF-7, MDA-MB231, PANC-1, and SiHa) using doses ranging from 0–60 nM after 48 h of incubation. In vitrocytotoxic effect of colchicine was observed in a dose-dependent manner, upto 40 nM in MDA-MB231 and 80 nM inPANC-1, MCF-7, HCT116, SiHa, and A549. Purified colchicine showed the least cytotoxicity at low doses and at higher concentration it showed a plateau-shaped dose-response curve, suggesting that the cells became resistant to colchicine at higher concentrations (Figure 4A,B).

### 3.5. Effect of Colchicine on Cell Migration

**Figure 5:**
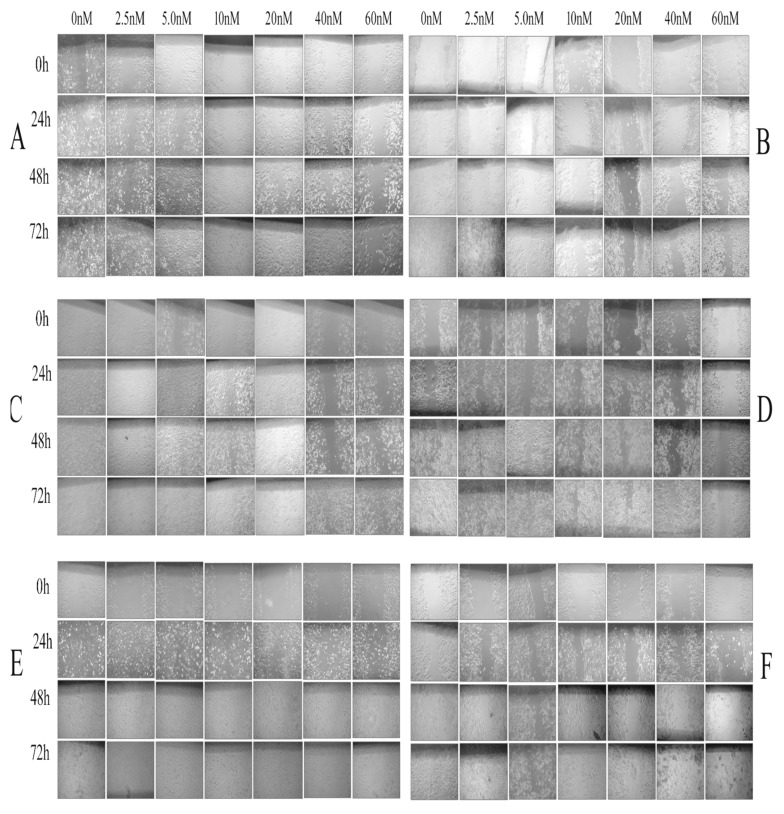
Phase contrast microscope images at 0, 24, 48, and 72 h post-scratch in six human cancer cell lines treated with varying doses of colchicine (2.5–60 nM). (**A**) A549, (**B**) HCT116, (**C**) SiHa, (**D**) PANC-1, (**E**) MDA-MB231, and (**F**) MCF-7.

**Figure 6:**
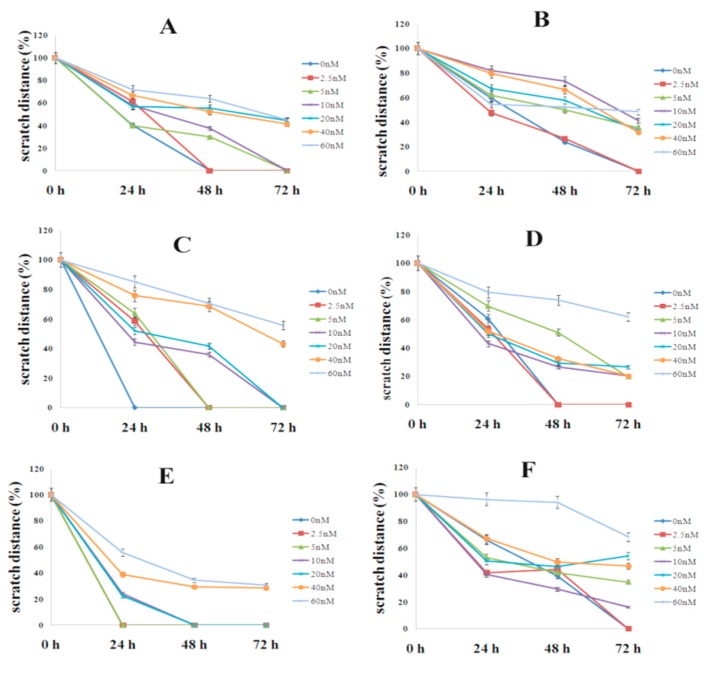
Antiproliferative effect of colchicine on cell migration. Cells were treated with varying doses of colchicine (2.5–60 nM) and the distance they moved was measured using a scratch assay at different time-points (0, 24, 48, and 72 h). Each line is labeled with the drug concentrations used in the experiment. (**A**) A549, (**B**) HCT116, (**C**) SiHa, (**D**) PANC-1, (**E**) MDA-MB231, and (**F**) MCF-7.

Cell migration was studied using in vitro scratch assay in six human cancer cell lines over a period of 72 h (Figure 5). In the untreated cells, the scratch area was closed at 24 h inMDA-MB231 and SiHa;48 h in A549 andPANC-1; and 72 h in HCT116 and MCF-7. Cells treated with low concentration of colchicine (2.5 nM) covered the scratch area at 24 h in MDA-MB231; 48 h in A549; SiHa and PANC-1; and 72 h in HCT116 and MCF-7 (Figure 5A–F).The reduced rate of cell migration was noticed with 5–20 nM concentration and the antiproliferative effect of colchicine was observed at 40 and 60 nM of colchicine, even 72 h post-scratch, in all studied cell lines (Figure 6A–F).

### 3.6. Analysis of Cell Cycle

**Figure 7:**
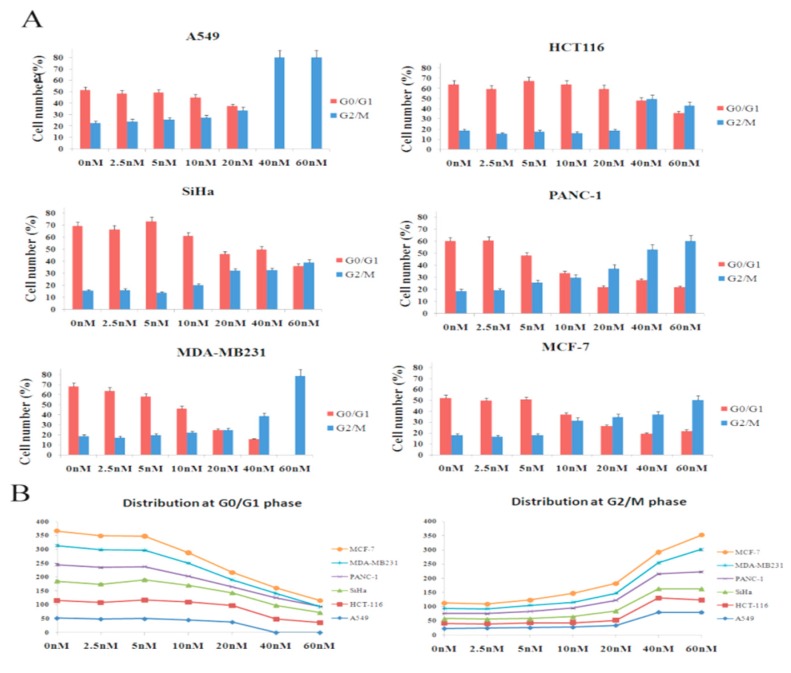
Effect of colchicine on cell cycle distribution in six human cancer cell lines (A549, HCT116, SiHa, PANC-1, MDA-MB231, and MCF-7). (**A**) Cells treated with different doses of colchicine (2.5–60 nM) for 24 h were stained with Propidium Iodide (PI) and analyzed using flow cytometry, based on 8000 cell counts. (**B**) Dose-response curve showing the effect of colchicine at the G0/G1 and G2/M phase, using doses ranging from 0 to 60 nM, using six human cancer cell lines.

The effect of colchicine on cell cycle progression was studied in six human cancer cell lines (Figure 7). The result demonstrated that untreatedA549 cells distributed 52% in G0/G1 (2C DNA), and 23% in the G2/M phase (4C DNA), whereas at higher concentrations of colchicine (40 and 60 nM), the cells showed mitotic arrest (80% in G2/M phase). At a 20 nM concentration, G0/G1 and G2/M peaks were almost equal. Untreated SiHa contained 69% cells in the G0/G1, and 16% in the G2/M phase (Figure 7). The mitotic arrest was initiated at 20 nM in SiHa, contained 46% cells in G0/G1, and 32% in the G2/M phase, while at 60 nM, the cells were distributed equally in both phases.

The untreated PANC-1 and MCF-7 cells showed a normal distribution in the cell cycle phase (PANC-1—60% G0/G1 and 19% G2/M, MCF-7—52% G0/G1 and 18% G2/M), while thetreated cells with 20–60 nM showed increasing4C (DNA Count; 4C) and decreasing 2C (DNA Count; 2C) content in both cell lines. Similarly, the untreated HCT116 cells distributed 64% in the G0/G1 and 19% in the G2/M phase within creasing 4C and decreasing 2C content at both concentrations (40 nM and 60 nM). In MDA-MB231, the control showed a normal cell cycle distribution; 69% of cells in the G0/G1 and 19% in the G2/M phase (Figure 7), whereas at higher concentrations of colchicine (60 nM), the cells showed mitosis arrest (G2/M phase). Increased 4C DNA and decreased 2C DNA content was observed at 40 nM, which indicated initiation of mitotic arrest at the same concentration.

## 4. Discussion

Considering the medicinal value of colchicine, we attempted to optimize colchicine extraction from *Gloriosa superba* seeds. Super Critical Fluid (CO_2_) extractor (SCFE) was used to obtain the crude seed extract (Figure 1). Therefore, the SCF extraction conditions (60 °C per 400 bar; flow rate—100 g/min of liquid CO_2_ with 3 g water as co-solvent) could be admitted as the best method to extract colchicine from the seeds of *Gloriosa* (Table 1). The temperature and pressure had significant influence on the extraction of colchicine. The crude seed extract was subjected to column chromatography for further purification. The isolated colchicine was quantified (99.82%) by HPLC. Colchicine extracted from *Gloriosa* through the solvent extraction method has already been reported [13,14,46]. Chemical composition screening of the *Gloriosa* seeds was performed using LC–MS QTof.

Colchicine possesses anticancer activity and the anticancer mechanism involves the colchicine–tubulin interaction, which disrupts microtubule dynamics [36,47,48,49]. It has been long used as a medicine for the treatment of gout and Familial Mediterranean Fever [29,30]. Recent studies have opened up the possibility of safe use of colchicine as an anti-cancer medicine with a controlled dose administration [47]. Colchicine causes high fatality after acute ingestions exceeding 0.5 mg/kg. The effective plasma concentrations ranges from 0.5 to 3 ng/mL and the toxic effects appear at a level of 3 ng/mL or above [49,50]. Our results demonstrated that purified colchicine was merely toxic at low concentrations and exhibited cell cytotoxicity in a dose-dependent manner up to a concentration of 40 nM in MDA-MB231, 80 nM in PANC-1, HCT116, MCF-7, SiHa, and A549.Concentrations higher than this give a plateau-shaped dose-response curve, indicating that the cells perhaps develop a resistance to colchicine [41]. Colchicine has been reported to play an anticancer role in lung cancer, at doses of 50 nM, and at doses of 100 nM in breast and liver cancer [41,47]. The significant antiproliferative effect of colchicine on cancer cell lines was observed at 40 nM and 60 nM concentrations 72 h post-scratch and at low concentrations (2.5 nM and 5 nM), it did not have any effect on antiproliferation (Figure 4).

Colchicine inhibits the progression from the G1 to the M phase (G2/M phase arrest) by disrupting the formation of the mitotic spindle required for mitosis [32]. It was reported that colchicine causedG2/M phase arrest at 50 nM concentrations in A549 [47]. In the present study, our result showed that colchicine caused mitotic arrest (G2/M phase) at 40 and 60 nM concentrations, in A549 and at 60 nM in MDA-MB231. The other three cell lines (SiHa, HCT116, and PANC-1) did not cause complete mitotic arrest at 60 nM, however, high G2/M (4C DNA) and low G0/G1peak (2C DNA) was observed, which indicated that mitotic arrest was initiated (Figure 5A,B). The observed cell-line-specific differences in the cell cycle progression from the G1 to the M phase with colchicine treatment might be due to the differences in drug uptake or other cell-type-specific drug effects. These findings indicated that colchicine was least toxic at low concentrations (concentrations included in the clinically acceptable range), but did not show any antiproliferative effect and caused no mitotic arrest. The uncontrolled rate of mitosis is aprominent feature of cancer cells, which makes them more vulnerable to mitotic poison than the noncancerous cells. The available reports suggest the use of colchicine at a dose of 2.5 nM is considered safe in lung cancer [47].

## 5. Conclusions

In conclusion, to avoid side effects of impurities present in colchicine, we developed methods of extraction and purification that produced a refined colchicine with high levels of activity, demonstrating its possible medicinal use. The present findings support that purified colchicine can be used in lung cancer, breast cancer, colon cancer, pancreatic cancer, and cervical cancer, because it was merely toxic at low concentrations and caused no mitotic arrest. Further pharmacological and clinical studies are required to establish these findings.

## Figures and Tables

**Table 1:** *Gloriosa superba* extraction and yield correlation.

Exp. No.	Gloriosa Seeds Loaded (Kg)	Pressure of Extractor	Temp (°C)	Flow Rate of Liquid CO_2_ (g/min) (3% Water as co Solvent)	Yield (G)	Assay of Colchicine (HPLC) (%)
1	3.0	200	60	100	1.2	Nil, waxy
2	3.0	250	60	100	5.0	3.0
3	3.0	300	60	100	10.3	6.92
4	3.0	350	60	100	56.2	20.3
5	3.0	400	60	100	72.8	27.0
6	3.0	450	60	100	80.1	16.15

**Table 2:** UPLC–MS QTof identified compounds of the Gloriosa superba seed in the positive and the negative mode.

S. No.	Component Name	RT (min)	Positive Mode		Negative Mode	
			Observed *m*/*z*	Response	Observed *m*/*z*	Response
1	Chelidonic acid	0.91	185.0079	3211	-	-
2	Adenosine	0.95	268.1031	151304	266.0877	1956
3	o-Coumaric acid	0.95	165.0537	23392	-	-
3	Vanillic acid	2.32	-	-	167.0337	2640
4	Vanillic acid β-D-glucopyranosyl ester	3.15	-	-	329.0864	9708
5	Catechin 7-O-β-D-glucopyranoside	4.58	453.1383	14809	451.1238	131,920
6	(−)-Epicatechin	5.28	291.0857	33690	289.0699	5584
7	Curculigoside B	5.28	453.1383	23748	451.1241	109,583
8	Caffeic acid	5.61	-	-	179.0335	4978
9	d-Isoboldine	5.97	328.1536	100400	-	-
10	Gentiatibetine	6.07	166.0851	6893	-	-
11	Procyanidin B7	6.28	-	-	577.1358	227,928
12	Daidzein	7.03	255.0659	4567	-	-
13	Colchicoside	7.11	548.2137	2177554	546.1986	213,123
14	2-demethylcolchicine	7.11	386.16	556495	384.1445	1,186,230
15	Catechin	7.21	291.0853	38106	289.0702	93,291
16	Isoastilbin	7.27	-	-	449.1079	28,723
17	6-Hydroxykaempferol	9.55	303.0498	3793	-	-
18	1,2-didemethyl colchicine	9.65	372.1443	322523	370.1283	27,794
19	Naringin	10.24	-	-	579.1736	27,936
20	Kaempferol-3-glucuronide	11.84	465.1023	7577	463.0871	8259
21	Quercetin-3-O-β-D-glucopyranoside	12.26	-	-	463.0877	231,855
22	Quercetin	12.29	303.0495	96085	-	-
23	N-Deacetyl-N-methylcolchicine	12.61	372.1788	7489	-	-
24	Isoperlolyrine 2	12.9	265.0964	82453	263.0809	2075
25	Colchiceine	13.12	386.1601	2336427	384.1448	1,907,075
26	Anthraquinone	13.2	209.0599	7183	-	-
27	Flavanonol	13.2	241.0854	6229	-	-
28	6-Hydroxykaempferol-3-O-glucoside	14.61	465.1025	3279	463.0876	3857
29	6-Methoxykaempferol-3-O-β-D-glucopyranoside	14.89	-	-	477.1037	35,244
30	Isoquercetin	16.12	465.1023	4261	-	-
31	2,3-didemthylcolchicine	16.66	372.1433	167107	370.1282	18,762
32	Gloriosine	17.03	386.1598	882243	384.1441	73,037
33	3-demethylcolchicine	18.4	386.1613	2596448	-	-
34	Colchicine	18.59	400.1744	2315151	-	-
35	Procyanidin A2	18.6	-	-	575.1203	51,849
36	n-deacetylcolchicine	18.66	358.1664	821243	-	-
37	Morin	19.1	-	-	301.0336	52,761
38	Luteolin	19.33	287.054	6150	285.0392	6471
39	Cornigerine	20.45	384.1434	438658	-	-
40	Futoenone	24.41	341.1379	739283	-	-
42	Balanophonin	28.24	357.1324	32373	-	-
